# Efficient Thermal Pose Estimation: Balancing Accuracy and Edge Deployment for Smart Home Activity Recognition

**DOI:** 10.3390/s26061774

**Published:** 2026-03-11

**Authors:** Gabriela Vdoviak, Tomyslav Sledevič, Vytautas Abromavičius, Dalius Navakauskas, Artūras Kaklauskas

**Affiliations:** 1 Department of Electronic Systems, Vilnius Gediminas Technical University, Saulėtekio Ave. 11, LT-10223 Vilnius, Lithuania; tomyslav.sledevic@vilniustech.lt (T.S.); dalius.navakauskas@vilniustech.lt (D.N.); 2 Department of Construction Management and Real Estate, Vilnius Gediminas Technical University, Saulėtekio Ave. 11, LT-10223 Vilnius, Lithuania

**Keywords:** pose detection, keypoints detection, thermal images, convolutional neural networks, Jetson GPU

## Abstract

This study investigates efficient thermal-image human pose estimation under edge deployment constraints for smart home activity recognition. A single-person thermal dataset of 2500 images was collected and annotated with 17 body keypoints. YOLO11-pose and YOLOv8-pose models were trained and evaluated across all five model scales (*n*–*x*) at three input resolutions 640 × 512, 320 × 256, and 160 × 128 px. The accuracy was evaluated using box mean Average Precision (mAP50–95), pose mAP50–95, and Object Keypoint Similarity (OKS) metrics. Runtime performance was assessed using per-image latency and power measurements on three NVIDIA Jetson platforms: Orin Nano 4 GB, Orin Nano 8 GB and AGX Orin 64 GB, using PyTorch and TensorRT at FP32, FP16, INT8 precision. Human detection remained consistently high across model variants, whereas pose accuracy decreased as the input resolution was reduced. TensorRT FP16 preserved pose accuracy relative to PyTorch and TensorRT FP32, with minimal changes in OKS and pose mAP50–95, while notably reducing per-image latency and power consumption. INT8 further reduced power consumption and in some configurations improved latency, but caused configuration-dependent losses in OKS and pose mAP50–95. The findings indicate that FP16 offers the best accuracy–efficiency balance for thermal pose estimation on edge devices, while practical feasibility depends on device capabilities and memory limitations.

## 1. Introduction

Human pose estimation represents the structure of the human body by identifying anatomical keypoints and is widely used for analyzing human behavior in visual data. Pose-based representations have been widely applied across diverse domains, including sports analysis, human–computer interaction, surveillance, and healthcare, enabling quantitative analysis of human motion and automated assessment of body movements. A prominent example in the healthcare domain is home physiotherapy and telerehabilitation, where pose estimation enables remote monitoring of therapeutic exercises and functional recovery, supporting clinical supervision outside traditional care settings [1,2,3]. In smart home environments, pose-based representations provide a concise description of posture and movement without reliance on detailed appearance information. Consequently, pose-based cues support tasks such as activity recognition [4], fall-risk assessment [5], and long-term monitoring of behavioral patterns [6]. However, deploying such systems in residential settings requires reliable, continuous operation under practical constraints, motivating systematic consideration of sensing and deployment choices [4,5].

In the context of smart home monitoring, thermal imaging has emerged as a privacy-preserving alternative to RGB cameras, which capture identity-revealing appearance information and are sensitive to illumination conditions [7,8]. By encoding human presence through temperature patterns rather than visual texture, thermal data supports behavioral analysis and reliable operation in low-light or dark environments. Concurrently, thermal imagery introduces specific considerations for pose estimation such as reduced spatial detail, thermal noise, and lower contrast between adjacent body regions [9]. These factors can influence joint separability, particularly for small or partially occluded body parts, and motivate the development and optimization of pose estimation approaches that remain robust under practical sensing and edge deployment conditions.

Pose estimation models can be deployed across a variety of computing platforms, including workstation- or server-based GPU systems and embedded devices with limited computational resources. Compared with server- or cloud-based processing, on-device inference reduces latency, avoids continuous video transmission, and improves robustness to network interruptions, while better aligning with privacy-preserving principles [10,11]. Embedded GPU platforms such as the NVIDIA Jetson support always-on operation due to their smaller footprint and lower power consumption compared to workstation-class systems. However, tight constraints on compute capability, memory, and energy mean that real-time performance depends strongly on model scale, input resolution, and numerical precision, underscoring the importance of hardware-aware optimization and on-device profiling [12].

Prior studies have investigated human pose estimation from thermal imagery, primarily by adapting existing pose estimation models to the characteristics of infrared data. Common strategies include supervision transfer from RGB-trained pose estimators [13], domain adaptation techniques [14], and the use of lightweight backbones with resolution-robust training procedures [15,16]. Additional robustness is often obtained through data augmentation [17], multi-modal or multi-view configurations [18]. Despite these advances, publicly available thermal datasets with reliable keypoint annotations remain limited compared to the RGB domain, particularly for data captured under realistic residential conditions [17]. This limitation complicates the systematic evaluation of pose estimation robustness and efficiency under deployment-oriented constraints relevant to smart home applications. Concurrently, several studies have examined the deployment of pose-based methods across different computing platforms, most commonly targeting low-cost CPU devices such as the Raspberry Pi or workstation- and server-class GPU systems [19,20,21]. Embedded GPU platforms such as NVIDIA Jetson have been explored more recently as energy-efficient edge deployment solutions [22,23]. However, existing studies often evaluate model performance on a single hardware platform or configuration and tend to emphasize either pose estimation accuracy or inference speed in isolation. As a result, there is limited evidence jointly characterizing pose accuracy and keypoint consistency alongside inference latency and power consumption across multiple embedded platforms using a unified thermal pose estimation pipeline.

The main aim of this study was to investigate efficient thermal pose estimation for smart home applications by systematically analyzing trade-offs between pose accuracy, inference speed, and energy consumption under deployment-realistic constraints. Compared to most existing studies that evaluate pose estimation performance or deployment efficiency in isolation, this study provides a unified analysis that combines a newly annotated thermal pose dataset with multi-platform edge deployment experiments. This analysis demonstrates how model scale, input resolution, and numerical precision affect both pose quality and real-time feasibility on embedded systems. The main contributions of this study are as follows:

- collected and annotated a single-person thermal pose dataset consisting of 2500 thermal images;
- conducted a systematic evaluation of thermal pose estimation models (YOLO-pose) under varying model scales and input resolutions;
- deployed trained models on three NVIDIA Jetson platforms, examining accuracy–efficiency trade-offs, inference speed, and power consumption.

## 2. Related Works

Recent work in human activity recognition has focused on privacy-preserving sensing modalities and on-device inference strategies. Thermal imaging and pose-based representations have been investigated as alternatives to RGB data for enabling activity analysis under privacy and illumination constraints [24,25]. Consequently, efficient deployment of human pose estimation models on embedded platforms has become an important consideration, as computational and power limitations influence real-time feasibility [26]. This section reviews prior contributions related to our work, focusing first on thermal-domain pose estimation and then on deployment of pose estimation methods on embedded platforms.

### 2.1. Thermal-Domain Pose Estimation for Privacy-Preserving Activity Analysis

Thermal pose estimation aims to extract human anatomical keypoints from infrared imagery, where appearance cues are limited and contrast is primarily determined by temperature differences. Although well suited to privacy-preserving smart home monitoring, thermal data presents challenges such as low resolution, sensor noise, and reduced joint separability under weak thermal contrast [9,27]. Some studies have also explored deep learning methods for enhancing perception and feature extraction from sensor data across different sensing modalities [28,29]. Such approaches highlight the growing role of learning-based techniques in improving robustness when sensor observations are noisy or contain limited structural information.

One strategy to address these challenges is supervision transfer from RGB data, enabling thermal pose learning without extensive manual annotation. Chen et al. [15] proposed ThermalPose, a bottom-up multi-person pose estimation framework trained using supervision transferred from paired visible imagery. The method achieved pose accuracy comparable to RGB baselines in well-contrasted scenes while remaining functional in dark environments. The authors further demonstrated that this lightweight model reduced computational complexity relative to standard OpenPose variants, although its performance decreased under occlusion and crowding. Similarly, Smith et al. [14] evaluated multiple pose estimation architectures adapted to thermal imagery and showed that top-down methods, particularly ViTPose, achieved higher accuracy than bottom-up approaches, but reduced throughput in multi-person scenarios. Beyond supervision transfer, lightweight and efficiency-oriented model design has also been explored to support practical deployment on embedded systems. Zang et al. [16] introduced LMANet, a lightweight multi-stage attention network for single-person keypoint detection in far-infrared imagery. By combining a MobileNetV3 backbone with channel and spatial attention, the model achieves a favorable accuracy–efficiency trade-off while maintaining real-time performance. These findings highlight the importance of architectural choices when thermal imagery resolution and contrast are limited.

Other studies explored extended sensing configurations to improve robustness under occlusion and ambiguous thermal signatures. Zhu et al. [6] proposed a two-stage framework for in-bed health monitoring that estimates 2D pose from thermal sequences and fuses aligned depth information to recover 3D joint positions. While the approach improves robustness under darkness and partial occlusions, occlusion from bedding and depth ambiguity remain challenging. In their study, Zhu et al. [30] introduced a dual-channel cascaded network for 3D pose estimation from a single infrared video, leveraging temporal context and thermal intensity cues to regress joint depth, achieving a mean 3D joint error below 21 mm, with mean joint estimation error of 18.9 mm.

Multi-view thermal camera setups have also been explored to improve robustness of pose estimation under occlusion and viewpoint ambiguity. Lupión et al. [18] proposed a privacy-preserving multi-view infrared framework for 3D pose estimation in smart home environments, transferring annotations from paired RGB images to train thermal-specific detection and pose models. On a dataset collected with three low-cost thermal cameras, the proposed detector achieved 99.58% detection accuracy, outperforming baseline YOLO on thermal images. However, the approach relies on multiple synchronized cameras, which may limit deployment flexibility in residential settings.

Chen et al. [31] examined recognition from ultra-low-resolution infrared thermopile imagery and observed that limited spatial resolution and small human–environment temperature differences can blur or erase hand contour details, making recognition difficult. Although the task focuses on hand gesture recognition rather than full-body pose estimation, the findings remain relevant for thermal-based human analysis. While reconstruction-based enhancement can improve recognition accuracy, it introduces substantial computational overhead, underscoring the trade-off between accuracy and efficiency when low-cost thermal sensing hardware is used.

### 2.2. Edge Deployment of Pose Estimation Models

Edge deployment of pose estimation models requires balancing pose detection accuracy with inference latency, throughput, and energy consumption under tight computational constraints. Prior studies highlight that hardware-aware optimization and on-device profiling are essential to sustain real-time performance in smart home systems [26].

Several studies have focused on optimizing pose estimation models for embedded GPU platforms, particularly NVIDIA Jetson devices. Kuzdeuov et al. [23] evaluated YOLO11-pose variants by converting PyTorch models to TensorRT on a Jetson AGX Orin 64 GB platform and comparing FP32, FP16, and INT8 precision. The results show that TensorRT significantly reduces inference latency, for instance reducing YOLO11n from 21.3 ms in PyTorch to 3.2 ms in FP16 TensorRT, while preserving pose accuracy. Although INT8 inference achieves the lowest latency, it introduces noticeable accuracy degradation, leading the authors to identify FP16 TensorRT as the most practical configuration for real-time thermal pose estimation on Jetson devices. Similarly, Wang et al. [22] proposed a lightweight skeletal temporal model for real-time activity recognition intended for deployment on resource-constrained platforms. The pipeline combines lightweight pose estimators with a compact LSTM-based temporal model. The authors report real-time performance on a workstation-class GPU (31.4 FPS) and CPU (25.3 FPS), while deployment on an NVIDIA Jetson Orin Nano achieves approximately 11.18 FPS in a multi-person setting.

Other works focused on CPU-based embedded platforms. Analia et al. [21] proposed a privacy-preserving long-lie detection system deployed on a Raspberry Pi 5 using low-resolution thermal imagery and pose-derived features. By extracting a reduced set of reliable keypoints using a lightweight MediaPipe Pose variant and applying temporal logic-based classification, the system achieves real-time operation with high detection performance. Wang et al. [32] presented an edge-oriented pose-based activity recognition system for Raspberry Pi 4B, incorporating lightweight YOLO-Pose models, temporal convolutional networks, and hardware-aware optimizations such as adaptive frame sampling and INT8 quantization. Experimental results showed that these optimizations increased throughput from 6.1 FPS to 8.5 FPS, while improving the F1-score for abnormal behavior detection from 0.86 to 0.88. At a higher system level, Zeng et al. [13] introduced ThermiKit, an edge-optimized LWIR analytics framework for privacy-preserving smart home and eldercare monitoring. The framework integrates lightweight modules for detection, pose estimation, and tracking under a shared backbone adapted through RGB-to-thermal supervision transfer. Designed to operate under a sub-1 TOPS compute budget, the system achieved real-time performance while improving detection by up to 6.42% mAP over baseline YOLO models across multiple thermal sensors.

Existing research demonstrates that thermal pose estimation enables privacy-preserving human activity analysis, but remains challenged by factors such as low spatial resolution, thermal noise, occlusion, and limited joint separability. Prior studies have proposed a variety of approaches to enhance robustness, such as supervision transfer, lightweight architectures, and multi-view or depth-assisted designs, often at the cost of increased computational complexity or reliance on constrained sensing setups. In regard to deployment, recent studies show that pose estimation can be executed on embedded platforms such as Raspberry Pi and NVIDIA Jetson through model compression, quantization, and hardware-aware optimization. However, existing implementations differ substantially in sensing modality, pose representation, optimization strategy, and evaluation metrics, which complicates direct comparison of practical performance limits. In particular, few works systematically evaluate inference speed, power consumption, or pose accuracy across multiple edge platforms using a consistent thermal pose estimation pipeline, motivating further investigation under realistic smart home constraints.

## 3. Materials and Methods

**Figure 1:**
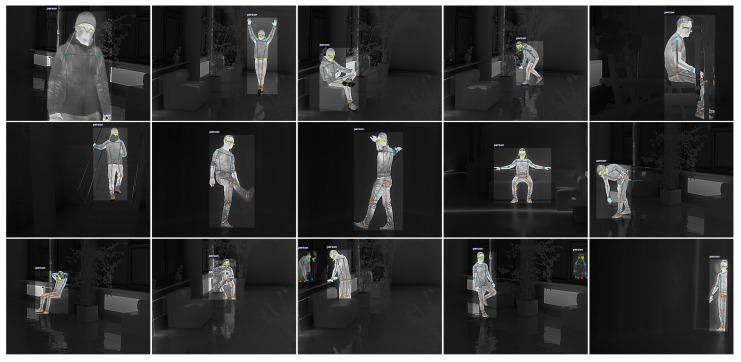
Example thermal images from the dataset with 17 annotated human pose keypoints.

The keypoint detection accuracy is evaluated using a newly collected single-person thermal image dataset. The dataset contains 2500 annotated thermal images with a spatial resolution of 720×576 px, acquired using a Lynx L15 thermal monocular (HIKMICRO, Hangzhou, China). Raw thermal video sequences were recorded in MP4 format at 25 fps. To reduce temporal redundancy, every fifth frame was extracted from the recordings and manually annotated. Each selected frame was labeled with a single-person bounding box and 17 body keypoints (Figure 1), following the standard human pose estimation annotation protocol.

To quantify the similarity between predicted and ground-truth keypoints, the keypoint similarity metric is defined as:

(1) $$KS_{i}=\exp\left(\frac{-d_{i}^{2}}{2s^{2}{\left(2\sigma_{i}\right)}^{2}}\right),$$

where $d_{i}$ denotes the Euclidean distance between the predicted and ground-truth location of the *i*-th keypoint, with i∈N=1,…,17. The parameter $\sigma_{i}$ represents the per-keypoint standard deviation, accounting for localization uncertainty, and *s* denotes the segmented area of the ground-truth object. In pose detection scenarios where only the bounding box width *w* and height *h* are available, the segmented area is approximated using a scaling factor of 0.53, i.e., s=0.53wh.

The object keypoint similarity (OKS) score aggregates the similarity of all visible keypoints and is computed as:

(2) $$OKS=\frac{\sum_{i=1}^{17}KS_{i}\;\delta \left(v_{i}>0\right)}{\sum_{i=1}^{17}\delta \left(v_{i}>0\right)},$$

where $KS_{i}$ is the keypoint similarity of the *i*-th keypoint, δ(·) denotes the indicator function that evaluates to 1 if the condition is satisfied and 0 otherwise, and $v_{i}$ is the ground-truth visibility flag for the *i*-th keypoint. Only keypoints labeled as visible or occluded are included in the OKS computation.

The standard deviation parameter $\sigma_{i}$ varies significantly across different body joints. Head-related keypoints (nose, eyes, and ears) are associated with smaller $\sigma_{i}$ values, reflecting their higher localization precision, whereas body and limb keypoints (shoulders, hips, knees, and ankles) exhibit larger $\sigma_{i}$ values due to increased positional variability:

(3) $$\begin{array}{cc} \sigma = & [0.026,\;0.025,\;0.025,\;0.035,\;0.035,\;0.079,\;0.079,\;0.072,\;0.072,\;0.062,\;0.062,\\ \; & 0.107,\;0.107,\;0.087,\;0.087,\;0.089,\;0.089]. \end{array}$$

**Figure 2:**
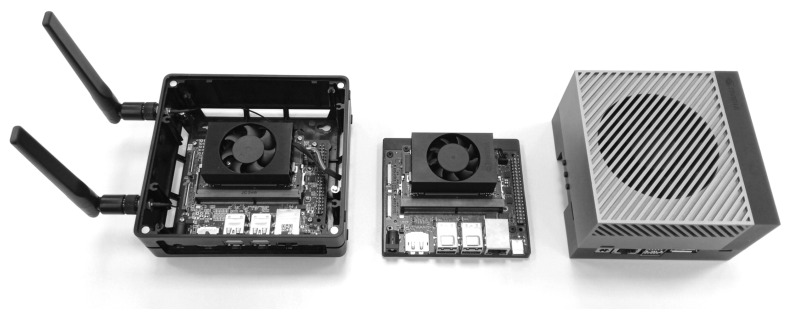
Jetsons (from left) Orin Nano 4 GB, Orin Nano 8 GB, AGX Orin 64 GB.

Three NVIDIA Jetson platforms were employed to investigate inference speed, power consumption, and numerical precision effects (PyTorch, FP32, FP16, and INT8): Jetson Orin Nano 4 GB, Jetson Orin Nano 8 GB, and Jetson AGX Orin 64 GB (Figure 2). These devices span a wide range of edge AI performance classes, enabling a systematic evaluation of efficiency-accuracy trade-offs. The Jetson Orin Nano modules feature an NVIDIA Ampere GPU with 512 CUDA cores and support configurable power modes of 10 W and 15 W, delivering up to approximately 20 TOPS (4 GB) and 40 TOPS (8 GB) of AI performance. The Jetson AGX Orin 64 GB integrates a significantly larger Ampere GPU with 2048 CUDA cores and 64 Tensor Cores, operating in configurable 30 W to 60 W power modes and providing up to 275 TOPS of peak AI performance. All platforms support hardware-accelerated mixed-precision and INT8 inference via Tensor Cores, allowing a consistent comparison of model latency, throughput, energy consumption and pose estimation accuracy across devices with substantially different computational resources.

Both YOLO11-pose and YOLOv8-pose models follow the single-stage detection paradigm of the YOLO family, in which object detection and keypoint localization are predicted directly from convolutional feature maps in a unified network. The architecture consists of a backbone for feature extraction, a neck that aggregates multi-scale features, and a task-specific head that jointly predicts bounding boxes and human pose keypoints. For pose estimation, the detection head is extended to regress the coordinates and confidence of 17 body keypoints in addition to the person bounding box. The YOLO11 and YOLOv8 pose variants mainly differ in architectural refinements and scaling strategies across model sizes (*n–x*), but follow the same overall design principle of pose prediction.

All YOLO-based pose estimation models were trained using an NVIDIA GeForce RTX 4080 Super GPU equipped with 16 GB of VRAM. The training environment consisted of Ultralytics v8.3.80, Python 3.12.9, PyTorch 2.5.1 and CUDA 12.6. Model training was performed at three fixed input resolutions: 640×512 px, 320×256 px, and 160×128 px. The dataset was partitioned into 80% for training and 20% for validation and testing, with an identical data split applied to all experiments to ensure comparability. To support reproducibility, a fixed random seed was used across all training runs, ensuring consistent data shuffling, weight initialization and augmentation behavior. Data augmentation included random image translation of up to ±10% of the image width, scaling with a gain of ±0.5 and horizontal flipping with a probability of 0.5. Mosaic augmentation was disabled during the final 10 training epochs to promote training stability.

Model optimization was performed using the AdamW optimizer with a learning rate of $1\times 10^{-3}$ momentum of 0.9 and weight decay regularization. The maximum number of training epochs was set to 1000, with model checkpoints saved every 10 epochs. Early stopping was enabled with a patience of 100 epochs, terminating training if no validation improvement was observed to prevent overfitting and reduce unnecessary computation. The batch size was dynamically adjusted between 4 and 96, depending on the input resolution and model complexity, to efficiently utilize available GPU memory. In practice, most models converged to their minimum validation loss within 200–400 epochs.

## 4. Results

This section presents the runtime and accuracy results of deploying YOLO11-pose and YOLOv8-pose models on three NVIDIA Jetson platforms. The results include inference time and power consumption measured across various model sizes, input resolutions, and numerical precisions. The object detection and pose estimation accuracy of the models is evaluated using mean Average Precision (mAP) and Object Keypoint Similarity (OKS).

### 4.1. Power Consumption and Inference Speed

This subsection reports inference-time power consumption and latency across model scales, input resolutions, precision modes and Jetson platforms.

**Figure 3:**
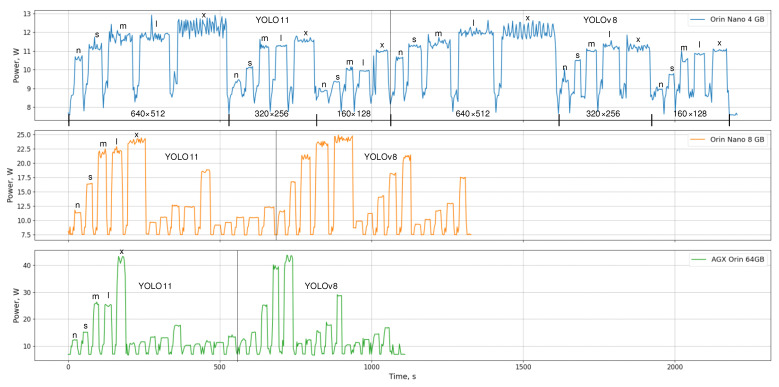
Power consumption of the Jetson platforms during inference with all YOLO11-pose and YOLOv8-pose PyTorch models at different input resolutions, with a 10 s idle pause between consecutive inference runs. The letters n–x denote model sizes: nano, small, medium, large, and extra-large.

Figure 3 presents the power consumption measured during inference on the test split for the three Jetson platforms while executing all YOLO11-pose and YOLOv8-pose PyTorch models. For each device, inference was performed sequentially across the five model sizes (*n*, *s*, *m*, *l*, *x*) at three input resolutions (640×512 px, 320×256 px and 160×128 px), first for YOLO11 and subsequently for YOLOv8 models. To ensure stable and repeatable measurements, a 10 s idle pause was inserted between consecutive inference runs, which is visible in the power traces as low-consumption intervals.

On the Orin Nano 4 GB, power consumption remained relatively stable, typically ranging between approximately 9–13 W, with modest stepwise increases for larger models and higher input resolutions. The Orin Nano 8 GB exhibited a wider dynamic range, reaching peak values of approximately 25 W for the largest models at the highest resolution. In contrast, the AGX Orin 64 GB showed the highest power variability and peak consumption, exceeding 43 W for the largest (*x*) models, reflecting its substantially higher computational capacity. Across all platforms, increasing model size and input resolution resulted in longer processing times and higher sustained power consumption, while smaller models and lower resolutions produced shorter inference durations with reduced power peaks.

A clear distinction across platforms is that the devices operate in different power regimes as workload increases. The Orin Nano 4 GB remains within a narrow power band, indicating limited headroom to raise instantaneous power with increasing model scale and input resolution. Consequently, higher workloads are expressed primarily as longer execution time rather than substantially higher peaks. In contrast, the Orin Nano 8 GB, and particularly the AGX Orin 64 GB, exhibit larger increases in power draw for heavier configurations, which reduces total completion time at the expense of higher power demand. These trends highlight that deployment decisions should consider power and runtime jointly, because energy per inference is determined by the combined effect of power draw and execution duration rather than by peak power alone.

**Figure 4:**
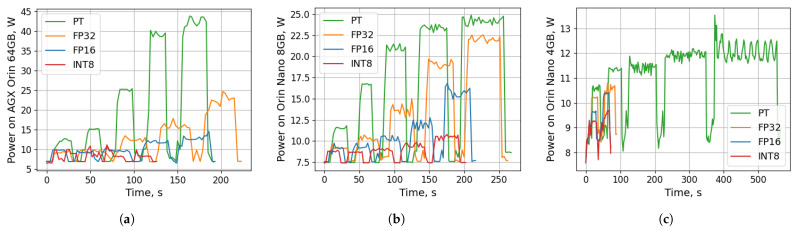
Power consumption during inference with YOLOv8-pose models (*n*, *s*, *m*, *l*, *x*, respectively with a 10 s pause between consecutive inference runs) and numerical precisions (PyTorch, FP32, FP16, and INT8) at 640×512 px input resolutions on Jetson platforms: (**a**) AGX Orin 64 GB, (**b**) Orin Nano 8 GB, (**c**) Orin Nano 4 GB (only *n* and *s* model variants).

Figure 4 summarizes the temporal power behavior of all YOLOv8-pose model variants during repeated inference on three Jetson platforms. Each model is executed sequentially with a 10 s idle interval to clearly distinguish individual runs. On the AGX Orin 64 GB (Figure 4a), the larger models (*l* and *x*) exhibit pronounced peaks approaching 40–44 W, while the smaller variants maintain substantially lower and more stable power envelopes. The separation between precision modes is most evident for the larger variants, where PyTorch produces the highest peaks, whereas FP16 and INT8 remain substantially lower. The Orin Nano 8 GB (Figure 4b) follows the same trend but with reduced magnitude, typically operating within 8–25 W depending on model size and precision mode, demonstrating a more constrained but still scalable power profile. For the largest variants, FP32 rises toward the upper end of the observed range, while FP16 and INT8 remain lower and exhibit reduced short-term fluctuations. The most resource-limited device, Orin Nano 4 GB (Figure 4c), can only deploy the *n* and *s* models due to insufficient memory for compiling the *m*, *l* and *x* engines. Despite this, it sustains comparatively modest power levels around 8–13 W with short, well-defined execution bursts separated by idle periods. For the supported *n* and *s* variants, PyTorch consistently draws the most power and shows the greatest temporal fluctuation. FP32 lowers the peak demand but remains noticeably above baseline. In contrast, FP16 and INT8 operate near the device baseline, with INT8 generally producing the lowest and smoothest power trace. Across all platforms, the precision mode has a visible effect: PyTorch and FP32 consistently draw the highest and most fluctuating power, whereas FP16 and particularly INT8 deliver more energy-efficient inference, underscoring the benefit of reduced numerical precision for edge deployment.

Table 1 reports per-image latency and power consumption for YOLO11-pose and YOLOv8-pose models on the Jetson AGX Orin 64 GB across three input resolutions and four precision modes. For both architectures, per-image time increases with model scale and decreases with input resolution. For instance, under FP16 precision mode, YOLOv8 latency ranges from 19.0–51.3 ms at 640×512 px, 10.5–30.2 ms at 320×256 px, and 8.5–20.3 ms at 160×128 px (from *n* to *x*). At 640×512 px, the *n* and *s* variants remain below 30 ms in FP16 and INT8, whereas the *l* and *x* variants increase to approximately 40–57 ms depending on precision. Across resolutions, YOLO11 and YOLOv8 exhibit comparable latency trends, with differences becoming most apparent for the larger model scales. The power measurements show a strong dependence on both model size and precision mode. PyTorch execution shows the highest power draw and the largest variation with model scale, reaching up to 42–43 W for the largest variants at 640×512 px. In contrast, the lower-precision TensorRT engines operate in a substantially lower power band. FP16 typically draws about 9–13 W, and at resolution of 640×512 px its power increases gradually with model scale. INT8 precision mode is the most stable, remaining close to approximately 8–10 W across model sizes and resolutions. Lower power does not always translate into the lowest latency. While INT8 generally minimizes power, FP16 is faster for several model–resolution pairs, with only a small increase in power draw. At 640×512 px, FP32 does not reliably improve latency for the larger models and can be slower than PyTorch.

In Table 2, the Jetson Orin Nano 8 GB results show clear latency scaling with both input resolution and model size for YOLO11-pose and YOLOv8-pose models. At 640×512 px resolution, FP16 precision mode latency ranges from 25.4–56.3 ms for YOLO11 and 24.6–60.9 ms for YOLOv8 (from *n* to *x*), while INT8 precision mode reduces this to 23.2–52.7 ms and 22.3–54.2 ms, respectively. At 320×256 px resolution, FP16 precision mode spans 14.2–37.2 ms (YOLO11) and 13.1–42.2 ms (YOLOv8), with INT8 precision mode reaching 13.0–31.8 ms and 9.4–26.5 ms. At the lowest resolution, INT8 achieves the smallest latencies overall, down to 6.5 ms for YOLOv8-*n*, while remaining below 20 ms for both architectures up to the largest variant. Power consumption on the Orin Nano 8 GB remains comparatively constrained. At 640×512 px resolution, PyTorch draws the highest power and increases with model scale, reaching 23.9–24.5 W for the *x* variants, whereas FP16 typically remains within 9–16 W and INT8 stays near 8.7–10.3 W. At 320×256 and 160×128 px resolutions, FP16 and INT8 power consumption becomes nearly flat across model scales, remaining close to the device baseline while maintaining reduced latency relative to PyTorch and FP32.

Table 3 highlights the limitations and efficiency characteristics of the Jetson Orin Nano 4 GB for YOLO11-pose and YOLOv8-pose models. The missing entries indicate configurations where FP32, FP16, INT8 engines could not be generated, so only the *n* and *s* variants are available in optimized precision modes, whereas larger variants (*m*, *l*, *x*) are reported only for PyTorch execution. Within the supported variants, reduced-precision execution substantially lowers latency compared with PyTorch. For example, at resolution of 640×512 px, YOLOv8-*n* decreases from 43.2 ms (PyTorch) to 25.7 ms (FP16) and 22.5 ms (INT8), and YOLOv8-*s* decreases from 70.1 ms (PyTorch) to 29.2 ms (FP16) and 25.6 ms (INT8). Reducing the input resolution further lowers latency, with YOLOv8-*n* reaching 14.8 ms at 320×256 px and 8.8 ms at 160×128 px in INT8. Power consumption on the Orin Nano 4 GB remains comparatively stable. At resolution of 640×512 px, PyTorch reaches approximately 10.5–12.2 W depending on the model variant, while FP16 and INT8 operate in a narrower band around 9.1–9.6 W for the supported *n* and *s* configurations. At 320×256 and 160×128 px resolutions, FP16 and INT8 power draw remains close to the device baseline (approximately 8.5–9.0 W), with only minor variation between *n* and *s*. Overall, the table shows that lower-precision modes reduce latency while keeping power consumption low and stable on the 4 GB platform for the configurations that fit in memory.

Comparing across all evaluated platforms, power consumption and latency scale consistently with computational performance. The AGX Orin 64 GB exhibits the highest power usage but also the best inference speed, making it suitable where maximum throughput is required. The Orin Nano 8 GB operates in a mid-range regime, consuming noticeably less power while still delivering reasonable latency across a wider range of models. The Orin Nano 4 GB shows the lowest overall power levels, but it is also the most constrained platform. Optimized FP32, FP16, and INT8 engines are available only for the *n* and *s* variants. Across all tested devices, reduced precision (especially INT8) generally lowers power consumption relative to PyTorch and FP32, while FP16 and INT8 typically provide substantial latency reductions compared with full precision. However, latency gains are not strictly monotonic across all configurations, and FP16 can be faster than INT8 for certain model-resolution combinations. These findings show the importance of reduced-precision inference for efficient deployment on edge hardware.

### 4.2. Precision and Keypoint Similarity

This subsection examines how numerical precision and input resolution influence thermal pose estimation quality, with emphasis on keypoint consistency under edge-deployment constraints. It is important to note that detection and keypoint localization respond differently to reduced precision and spatial downsampling. Therefore, both detection-oriented and pose-oriented metrics are considered in order to differentiate between missed detections and localization errors.

Table 4 shows detection and pose estimation accuracy for YOLO11-pose and YOLOv8-pose across five model scales (*n*–*x*), three input resolutions, and four numerical precision modes. For PyTorch, TensorRT FP32, and TensorRT FP16, box mAP50–95 remains high across all configurations, with only modest reductions at the lowest resolution. In contrast, pose accuracy exhibits a stronger dependence on spatial resolution: both pose mAP50–95 and mean OKS decrease as the input is downsampled, reflecting the loss of fine-grained thermal detail needed for reliable keypoint localization. Across both model families, FP16 closely matches PyTorch and FP32, with differences typically confined to the third decimal place, indicating that mixed-precision inference preserves both detection and pose accuracy.

INT8 precision mode shows a different behavior. While box mAP50–95 generally remains relatively high, pose quality can degrade substantially and the extent of degradation depends on the specific configuration. At 640×512 px resolution, FP16 pose mAP50–95 ranges from 0.892–0.935 for YOLO11 and 0.900–0.942 for YOLOv8, whereas INT8 drops to 0.136–0.331 and 0.279–0.430, respectively. The same pattern is reflected in mean OKS, which remains high for FP16, up to 0.940 for YOLO11-*x* and 0.945 for YOLOv8 at 640×512 px, but decreases markedly for INT8 in several model variants. Notably, INT8 pose metrics do not follow a strictly monotonic trend with resolution, suggesting that quantized pose accuracy is sensitive to the interaction between model capacity, input resolution, and calibration. The results identify FP16 as a robust deployment setting for preserving pose accuracy, whereas INT8 should be applied selectively when efficiency constraints dominate and the resulting keypoint quality is acceptable for the downstream task.

Pose accuracy stability under reduced precision is summarized in Table 5 using the mean and standard deviation of the accuracy degradation relative to PyTorch, averaged across five model scales at each input resolution. Across both YOLO11 and YOLOv8, FP32 and FP16 produce near-zero changes in both ΔOKS and ΔPose mAP50–95, with deviations on the order of $10^{-3}$ and standard deviations of approximately 0.001–0.002. This indicates that the TensorRT FP32, FP16 engines preserve pose accuracy consistently across model scales and resolutions, with no practically meaningful loss relative to PyTorch.

In contrast, INT8 precision mode introduces a substantial and resolution-dependent degradation in both metrics. For YOLO11, the mean ΔOKS decreases from −0.327±0.081 at 640×512 px to −0.227±0.057 at 320×256 px and −0.165±0.042 at 160×128 px. The corresponding ΔPose mAP50–95 follows the same trend (−0.301±0.073 to −0.206±0.051 and −0.152±0.039). YOLOv8 exhibits closely matching behavior, with slightly smaller but comparable INT8 losses. The larger INT8 standard deviations relative to FP16 and FP32 suggest increased variability across model scales, consistent with quantization sensitivity to network capacity and configuration. These results confirm that FP16 offers a robust and architecture-independent optimization for edge deployment, whereas INT8 accuracy degradation is both model- and resolution-dependent and should be applied selectively.

**Figure 5:**
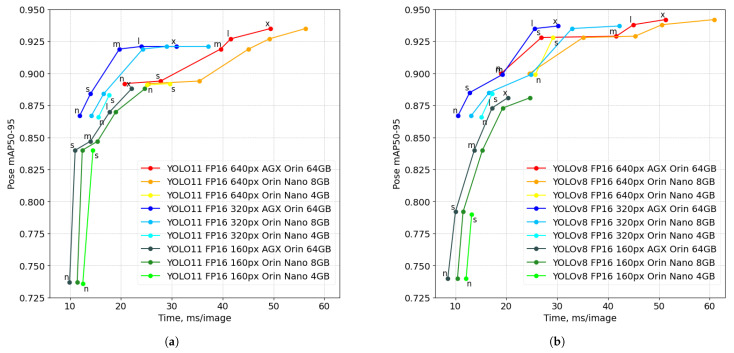
Precision vs. latency trade-off on Jetson platforms: performance comparison of YOLO11 (**a**) and YOLOv8 (**b**) FP16 engines on three Jetson platforms at three input resolutions (640×512 px, 320×256 px and 160×128 px). Horizontal axis represents per-image time for preprocessing, inference and postprocessing.

Figure 5 illustrates the relationship between inference latency (time per image) and pose mAP50–95 accuracy for YOLO11 and YOLOv8 FP16 engines across three Jetson platforms and three input resolutions. Each polyline corresponds to a fixed platform and resolution, with markers annotated by model scale (*n*–*x*). A consistent trade-off is observed: higher resolutions and larger models increase pose accuracy but also increase per-image time, whereas lower resolutions reduce latency at the expense of keypoint precision. The resolution setting primarily shifts the operating point range along both axes. At 160×128 px, most configurations cluster at low latency (roughly ∼10–20 ms/image) with lower pose mAP50–95 (approximately ∼0.74–0.89). At 320×256 px, latency increases (about ∼12–40 ms/image), while accuracy rises into the ∼0.87–0.94 range depending on model size. At 640×512 px, the highest accuracies are achieved (up to ∼0.94–0.95), but latencies extend to the upper end of the plotted range (up to ∼60 ms/image). Across all resolutions, the Jetson AGX Orin 64 GB occupies the leftmost region, indicating the lowest latency at comparable accuracy. The Orin Nano 8 GB follows the same accuracy trend but at higher per-image times, and the Orin Nano 4 GB is further constrained, with points concentrated on the smaller variants visible in the plot. For both architectures, accuracy gains tend to diminish for the largest scales: moving from mid-sized to large models increases latency more than it increases pose mAP50–95, particularly at the highest resolution.

**Figure 6:**
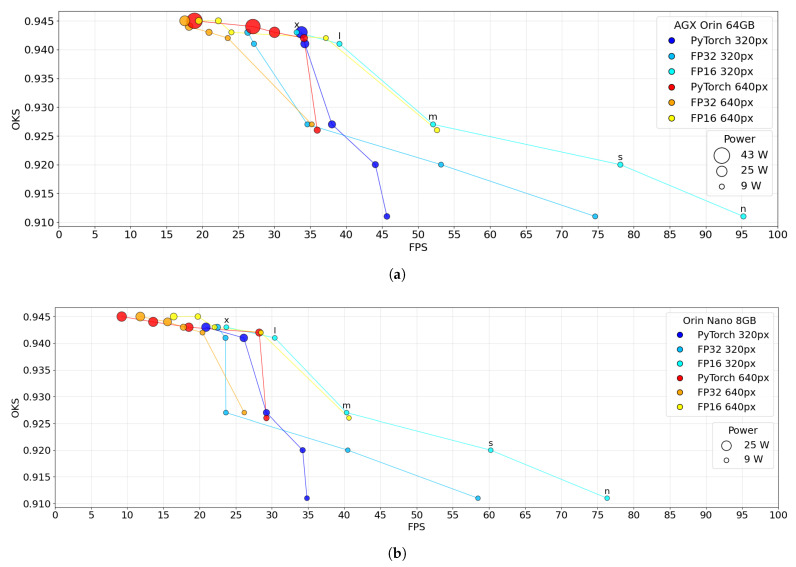
Object keypoint similarity (OKS) vs. speed trade-off on (**a**) Jetson AGX Orin 64 GB and (**b**) Jetson Orin Nano 8 GB platforms running YOLOv8 models.

Figure 6 shows the trade-off between pose estimation accuracy (OKS) and throughput (FPS) for YOLOv8 models evaluated on the Jetson AGX Orin 64 GB and Orin Nano 8 GB platforms. On both devices, larger model variants and higher input resolutions cluster toward higher OKS values but achieve lower FPS, indicating that improved keypoint localization accuracy is obtained at the expense of reduced throughput. These high-accuracy operating points are also associated with increased power demand, particularly on the AGX Orin 64 GB, where larger models at high resolution reach the upper range of the device’s power envelope. Conversely, smaller network variants and lower input resolutions achieve higher FPS with lower power consumption, but at the cost of reduced OKS, reflecting decreased localization precision under limited spatial detail. The AGX Orin 64 GB attains higher FPS than the Orin Nano 8 GB at comparable OKS levels, but with higher peak power usage, whereas the Orin Nano 8 GB provides a balanced compromise between accuracy, throughput, and power. Across both platforms, FP16 points generally lie to the right of FP32 and PyTorch at comparable OKS, indicating higher FPS for similar keypoint similarity. At 640×512 px resolution, OKS values are tightly clustered near the top of the axis across precision modes, while FPS varies more strongly, so differences are dominated by throughput and power rather than accuracy. At 320×256 px resolution, the smallest variants achieve the highest FPS, whereas moving toward larger variants increases OKS only slightly but shifts operating points leftward, reflecting diminishing accuracy returns for a substantial loss in throughput. Marker sizes increase for lower-FPS, high-resolution configurations, indicating higher power consumption. This effect is more pronounced on the AGX Orin 64 GB, where the marker-size range is wider and the highest-power points coincide with the largest models at 640×512 px resolution.

**Figure 7:**
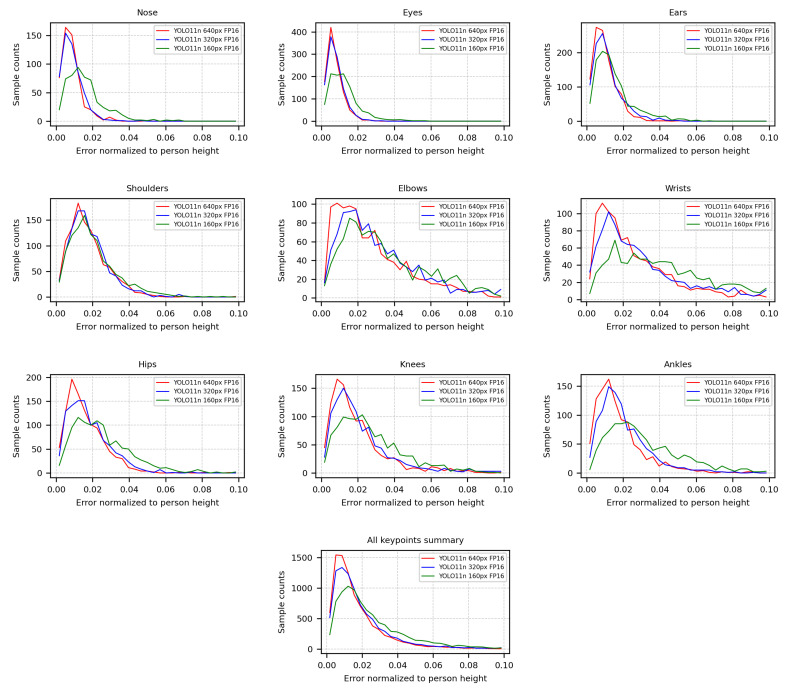
Distribution of keypoint localization error normalized to person height as a function of the number of samples, for different input resolutions.

Figure 7 presents the normalized absolute localization error of individual keypoints relative to person height, shown as error distributions for each input resolution. The y-axis reports sample counts, indicating how frequently a given normalized error occurs across the evaluated test instances. Across keypoints, the distributions are right-skewed, with most samples concentrated at low errors and a decreasing tail toward larger deviations. As expected, higher input resolutions consistently yield tighter distributions and shorter tails, while the lowest resolution exhibits broader spreads and more frequent large-error outliers. The resolution effect is smallest for larger, centrally located joints (shoulders and hips), where the peaks remain close and the main change is a modest tail increase. In contrast, distal joints (wrists, knees, and ankles) show the most pronounced degradation at 160×128 px, with visibly heavier tails extending toward 0.08–0.10 of person height. Head keypoints (nose, eyes, and ears) remain the most precisely localized in overall, with sharp peaks near small errors for 640×512 px and 320×256 px. The aggregated keypoints figure confirms the same ordering across resolutions, where 320×256 px stays close to 640×512 px, while 160×128 px shifts probability mass toward higher errors. These findings reinforce the importance of selecting an appropriate input resolution depending on the targeted application. High-precision tasks benefit from increased resolution, whereas coarse pose estimation remains feasible under strong resolution constraints.

**Figure 8:**
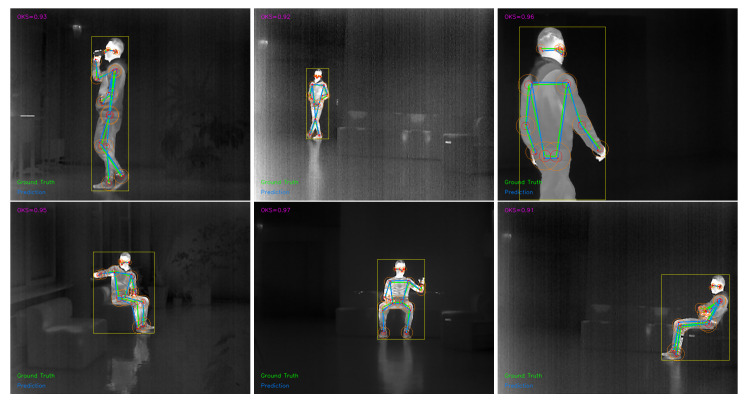
Qualitative pose estimation results on thermal images, illustrating OKS-based keypoint similarity. Ground-truth keypoints are shown in green and predicted keypoints in blue. The inner and outer circles indicate OKS similarity thresholds of 0.95 and 0.75, respectively.

In Figure 8 we provide a qualitative visualization of pose estimation results on thermal images, using the mean OKS score as an overall similarity indicator. Ground-truth keypoints are shown in green and predictions in blue, and each keypoint is accompanied by two concentric similarity regions. The inner and outer circles correspond to OKS similarity levels of 0.95 and 0.75, respectively, and the annotated mean OKS value summarizes the per-frame agreement between the two skeletons. The six examples cover different viewpoints and subject scales within the scene, allowing visual inspection of model behavior under typical indoor conditions. Across the six examples, the reported mean OKS values range from 0.91 to 0.97, indicating consistently strong agreement between predictions and ground truth. Higher-scoring cases occur when the person occupies a larger image region and limb contours are clearly separated from the background, which reduces ambiguity during keypoint placement. The lower end of the range is observed in more challenging frames, such as smaller or more distant subjects and seated poses with self-occlusion or interaction with furniture, where distal joints are harder to resolve. In these cases, visible offsets most often appear at the wrists and ankles, while the torso and proximal joints remain comparatively stable. These findings show that the proposed models are capable of reliable and anatomically consistent pose reconstruction in thermal imagery, supporting their suitability for smart-home human activity recognition scenarios.

## 5. Discussion

This study provides a unified evaluation of thermal pose estimation accuracy and deployment efficiency by combining a newly annotated single-person thermal dataset with on-device latency and power measurements on three NVIDIA Jetson platforms. The results show that bounding-box detection in thermal data is consistently high across model scales and resolutions (Table 4). In contrast, pose estimation remains the limiting factor because keypoints require finer spatial localization, which is challenging in thermal imagery under limited spatial detail and weak joint separability [27]. This separation is important for smart-home activity recognition, where downstream performance is often constrained by keypoint stability rather than person detection [4].

For both YOLO11-pose and YOLOv8-pose models, FP16 inference achieves accuracy comparable to PyTorch and FP32, while offering substantial reductions in latency and power consumption (Table 4 and Table 5). The mean accuracy difference remains close to zero, and the standard deviations across model scales are minimal, indicating stable numerical behavior under mixed precision. These findings align with prior Jetson-focused studies that identify TensorRT FP16 as a strong operating point for real-time thermal pose estimation [23], and with broader evidence that hardware-aware optimization and on-device profiling are essential for embedded deployment feasibility [12]. From a deployment perspective, FP16 represents a reliable operating point, combining stable accuracy with reduced power consumption when TensorRT conversion is feasible.

Across devices and model scales, INT8 quantization reduces power consumption compared with PyTorch and FP32, and it often shortens per-image latency for the same model and resolution (Figure 3 and Figure 4, Table 1, Table 2 and Table 3). However, the accuracy results show that quantization can substantially lower pose mAP50–95 and OKS metrics, particularly at higher resolutions and for several model variants (Table 4). This behavior is consistent with prior Jetson thermal pose evaluations that report INT8 speedups accompanied by measurable accuracy loss [23], and with edge pipelines that treat quantization as a throughput lever that must be balanced against task requirements [32]. The mean INT8 accuracy difference also shows greater variability across model scales (Table 5), which suggests that quantization sensitivity depends on network capacity and internal activation statistics. Importantly, INT8 does not always achieve the lowest latency, implying that memory traffic and precision-specific scheduling can dominate performance for certain model–resolution pairs. For smart-home applications, INT8 is therefore best viewed as an energy-saving option that requires per-configuration validation, especially when the target activities depend on accurate joint localization. These findings suggest that simple post-training INT8 quantization may be insufficient for pose estimation tasks that require fine-grained joint localization. More advanced approaches such as quantization-aware training, improved calibration with representative thermal samples, or selective or mixed-precision deployment could help reduce accuracy loss while retaining most of the efficiency benefits. These directions were not explored in the present study but represent promising future work for improving INT8 viability on edge devices.

Beyond numerical precision, input resolution and model scale define a consistent accuracy–latency trade-off across devices and model families (Figure 5). Higher resolutions and larger models improve pose accuracy, but they also increase per-image processing time and typically power consumption. The keypoint error distributions (Figure 7) indicate that distal joints experience the largest loss of localization precision at 160×128 px, which is consistent with weaker thermal boundaries and reduced discriminability for small structures under low spatial detail [31]. In contrast, central joints (shoulders and hips) remain comparatively stable across resolutions, suggesting that coarse posture cues can still be extracted reliably under strong downsampling. This supports a practical deployment guideline: low resolution can be sufficient for coarse activities (standing, sitting), while higher resolution is preferable when fine-grained motion cues or limb interactions are required.

While the Jetson AGX Orin 64 GB achieves the lowest per-image latency at comparable accuracy, it also reaches the highest peak power for larger variants at high resolution (Table 1, Figure 3 and Figure 4). The Orin Nano 8 GB provides a middle ground, maintaining moderate latency with a more constrained power profile (Table 2). On the Orin Nano 4 GB, memory limits prevented building optimized engines for several larger variants (Table 3), although PyTorch execution remained possible. This distinction matters for real deployment because the most efficient operating points often require TensorRT compilation and sufficient memory headroom, as also reflected in prior Jetson deployment studies that rely on TensorRT engines for real-time performance [23]. Therefore, hardware selection should consider not only achievable FPS, but also whether the intended model family and precision mode can be deployed reliably within the device memory budget.

In highly memory-constrained edge environments such as the Jetson Orin Nano 4 GB, several strategies may help mitigate memory bottlenecks during deployment. For instance, selective layer partitioning or layer-wise execution strategies can distribute intermediate activations across available memory regions, reducing peak memory demand during inference. In heterogeneous edge systems, pipeline parallelism between CPU and GPU components may also be used to overlap computation and memory transfers, enabling more efficient utilization of limited device resources. Additionally, model compression techniques such as structured pruning, lightweight backbone substitutions, or reduced activation precision may further lower the memory footprint while preserving acceptable accuracy. Although these approaches were not explored in the present study, they represent promising directions for extending thermal pose estimation pipelines to devices with tighter memory constraints.

The qualitative examples confirm that the predicted poses remain anatomically plausible and consistent across typical indoor scenes, with mean OKS values in the 0.91–0.97 range (Figure 8). Residual errors are most visible at the wrists and ankles in more challenging cases, such as smaller subjects or seated poses with self-occlusion or interaction with furniture. This pattern is consistent with the known difficulty of localizing distal joints in low-detail thermal imagery. For activity recognition, these error patterns suggest that models can provide reliable global pose structure and limb connectivity, while the precise localization of hand- and foot-related joints remains a dominant source of uncertainty. This observation supports the use of pose-derived features that are robust to small distal-joint offsets, such as torso orientation, hip–knee angles, or temporal smoothing of limb trajectories, especially under low-resolution operation [21].

This study uses a single-person thermal dataset acquired under a specific thermal sensor and indoor setup, which may limit generalization to multi-person scenes, different room layouts, or sensors with different noise and contrast characteristics [17]. The frame extraction strategy reduces temporal redundancy, but the dataset can still contain correlated viewpoints and backgrounds, and the evaluation does not explicitly test cross-subject or cross-environment transfer. Therefore, the reported accuracy–efficiency trade-offs should be interpreted as deployment-oriented evidence under controlled residential conditions rather than as a claim of broad domain generalization.

## 6. Conclusions

This study investigated efficient thermal pose estimation for privacy-preserving smart home activity recognition by combining a newly annotated single-person thermal dataset with deployment on three NVIDIA Jetson platforms: Orin Nano 4 GB, Orin Nano 8 GB, and AGX Orin 64 GB. A comprehensive evaluation was conducted to assess the accuracy, latency, and power trade-offs of the YOLO11-pose and YOLOv8-pose models across a range of model sizes, input resolutions, and numerical precision modes. The findings indicate that person detection in thermal imagery shows consistent robustness, while pose accuracy remains a limiting factor and is primarily affected by the quality of keypoint localization.

Across the two model families examined, TensorRT FP16 was identified as the most reliable option for edge deployment. FP16 maintains pose accuracy comparable to that of PyTorch and FP32, while concurrently achieving a substantial reduction in per-image latency and power consumption across all evaluated devices. INT8 has been shown to reduce power consumption and improve latency. However, it introduces substantial degradations in pose mAP50–95 and OKS. Consequently, INT8 is only suitable when energy savings are a primary concern and the resulting keypoint quality is validated for the specific target task. Input resolution and model size define a consistent accuracy–efficiency trade-off. Higher resolutions improve keypoint localization, while a high degree of downsampling increases distal joint errors (wrists, knees, and ankles) and reduces fine-grained pose fidelity. In addition, the selection of the embedded device affects not only throughput and power consumption but also which optimized engines can be deployed in practice, as memory limitations on the Orin Nano 4 GB restrict TensorRT support for larger model variants.

Future research could investigate thermal pose estimation under more diverse and realistic conditions, including multi-person scenes and occlusions to better assess generalization and improve distal-joint stability. In parallel, edge deployment could be extended with quantization-aware training and improved INT8 calibration to reduce pose degradation, together with memory-aware optimization strategies that enable larger models and higher resolutions on resource-limited Jetson devices.

## Figures and Tables

**Table 1:** Summary of the time per frame and power consumption of AGX Orin 64 GB. Per-image time includes preprocessing, inference and postprocessing. PT denotes PyTorch.

Model		Input	Time, ms				Power, W			
		Resolution	PT	FP32	FP16	INT8	PT	FP32	FP16	INT8
YOLO11-pose	n	640×512	29.6	30.6	20.7	20.2	12.3	9.3	9.7	9.6
	s		29.6	47.1	27.8	23.8	15.1	9.6	9.4	9.7
	m		33.0	46.9	41.6	34.7	25.7	12.8	9.9	9.8
	l		43.5	49.6	39.6	39.4	25.2	14.2	11.0	9.8
	x		49.0	56.9	49.4	42.2	42.7	22.5	13.4	9.8
	n	320×256	26.4	15.1	11.9	15.3	10.7	9.5	9.6	9.3
	s		26.9	19.7	14.0	16.5	11.6	9.5	9.9	9.3
	m		31.2	28.8	19.7	21.4	13.3	9.5	9.3	8.4
	l		42.5	34.8	24.0	25.0	13.0	9.5	9.4	8.2
	x		42.9	39.2	30.9	29.4	17.7	11.8	9.7	8.7
	n	160×128	25.0	12.1	9.9	10.3	10.2	9.2	9.4	9.3
	s		25.3	14.6	11.0	12.0	10.8	9.2	9.3	9.2
	m		30.1	19.1	14.0	15.7	11.5	8.7	9.7	8.7
	l		41.6	23.8	17.8	18.7	11.3	8.8	8.8	8.5
	x		41.8	31.0	22.1	23.8	13.5	9.2	9.4	8.3
YOLOv8-pose	n	640×512	27.8	28.4	19.0	19.3	12.6	9.2	9.8	9.6
	s		29.3	42.5	26.9	22.6	15.1	9.6	9.2	9.7
	m		33.3	47.8	41.6	33.3	25.2	12.4	9.6	9.8
	l		37.0	55.1	45.0	40.2	39.4	16.3	11.9	9.8
	x		52.9	57.0	51.3	42.4	42.9	23.5	13.4	9.8
	n	320×256	21.9	13.4	10.5	12.5	11.0	9.3	9.8	9.0
	s		22.7	18.8	12.8	14.0	12.3	9.2	9.7	8.7
	m		26.3	28.9	19.2	19.7	15.3	9.5	9.4	8.6
	l		29.2	36.8	25.6	23.4	17.8	9.8	9.5	8.4
	x		29.6	38.0	30.2	27.3	28.7	12.1	9.7	8.6
	n	160×128	20.6	10.4	8.5	9.3	10.4	9.3	9.7	9.3
	s		20.9	13.3	10.0	11.4	11.3	9.5	9.6	9.1
	m		25.1	19.2	13.7	15.3	12.4	9.3	9.7	8.6
	l		28.4	24.5	17.2	19.5	14.4	9.4	9.8	8.3
	x		29.0	30.4	20.3	21.7	16.8	9.5	9.7	8.4

**Table 2:** Summary of the time per frame and power consumption of Orin Nano 8 GB. Per-image time includes preprocessing, inference and postprocessing. PT denotes PyTorch.

Model		Input	Time, ms				Power, W			
		Resolution	PT	FP32	FP16	INT8	PT	FP32	FP16	INT8
YOLO11-pose	n	640×512	37.3	39.7	25.4	23.2	11.3	9.4	9.3	8.8
	s		38.2	47.7	35.4	29.0	16.4	10.2	9.4	8.7
	m		50.6	56.2	45.1	44.1	21.8	13.0	10.4	9.1
	l		60.8	57.6	49.2	46.7	22.1	16.5	10.8	9.3
	x		102.8	79.5	56.3	52.7	23.9	21.1	13.7	10.3
	n	320×256	34.3	17.6	14.2	13.0	9.7	9.1	9.3	8.8
	s		35.2	24.6	16.6	14.8	10.6	9.1	9.4	8.9
	m		41.1	38.0	24.3	23.6	12.7	9.6	9.2	8.8
	l		56.1	38.8	29.0	24.6	12.5	10.1	9.3	8.7
	x		55.0	45.4	37.2	31.8	18.8	11.8	9.8	8.9
	n	160×128	33.1	12.8	11.4	10.5	9.2	9.1	9.0	8.8
	s		33.8	15.9	12.4	11.2	9.7	9.1	9.3	8.9
	m		40.3	21.9	15.4	13.6	10.5	9.2	9.3	8.9
	l		54.7	27.0	19.0	16.4	10.5	9.1	9.3	8.7
	x		55.4	35.9	24.7	19.8	12.3	9.6	9.3	8.7
YOLOv8-pose	n	640×512	34.2	38.2	24.6	22.3	11.6	9.2	9.3	8.8
	s		35.4	49.0	35.1	28.3	16.8	10.3	9.4	8.7
	m		54.0	56.3	45.3	44.3	21.1	13.8	10.5	9.0
	l		73.6	64.2	50.6	52.6	23.6	19.1	12.0	9.0
	x		108.3	84.7	60.9	54.2	24.5	22.1	15.8	9.1
	n	320×256	28.7	17.1	13.1	9.4	9.9	9.2	9.3	8.8
	s		29.2	24.7	16.6	14.0	11.2	9.2	9.4	9.1
	m		34.2	42.3	24.8	20.2	14.1	9.7	9.3	8.7
	l		38.3	42.4	32.9	25.9	18.2	10.7	9.6	8.7
	x		47.9	44.5	42.2	26.5	21.2	13.4	9.8	9.1
	n	160×128	27.6	11.9	10.4	6.5	9.3	9.3	9.1	8.8
	s		28.3	14.8	11.5	10.5	10.2	9.2	9.3	8.9
	m		33.6	23.2	15.3	12.8	11.7	9.1	9.3	9.0
	l		37.9	29.1	19.3	15.6	13.0	9.4	9.2	8.8
	x		37.2	36.5	24.7	15.1	17.6	9.8	9.2	8.8

**Table 3:** Summary of the time per frame and power consumption of Orin Nano 4 GB. Per-image time includes preprocessing, inference and postprocessing. PT denotes PyTorch.

Model		Input	Time, ms				Power, W			
		Resolution	PT	FP32	FP16	INT8	PT	FP32	FP16	INT8
YOLO11-pose	n	640×512	43.7	31.9	25.1	24.4	10.6	10.0	9.3	9.2
	s		71.9	47.7	29.6	26.0	11.2	10.4	9.6	9.3
	m		135.9	-	-	-	11.7	-	-	-
	l		168.8	-	-	-	11.7	-	-	-
	x		292.2	-	-	-	12.2	-	-	-
	n	320×256	36.3	18.3	15.6	14.5	9.3	9.0	8.8	8.8
	s		37.0	21.9	17.7	16.0	10.1	9.0	9.0	8.8
	m		52.1	-	-	-	11.2	-	-	-
	l		65.7	-	-	-	11.2	-	-	-
	x		114.2	-	-	-	11.5	-	-	-
	n	160×128	34.8	14.3	12.5	13.3	8.9	8.7	8.5	8.6
	s		35.2	16.2	14.5	13.5	9.3	8.9	8.7	8.7
	m		41.0	-	-	-	10.0	-	-	-
	l		57.2	-	-	-	9.9	-	-	-
	x		69.8	-	-	-	11.0	-	-	-
YOLOv8-pose	n	640×512	43.2	31.6	25.7	22.5	10.5	9.6	9.1	9.1
	s		70.1	48.5	29.2	25.6	11.3	10.3	9.5	9.1
	m		136.9	-	-	-	11.4	-	-	-
	l		207.6	-	-	-	11.9	-	-	-
	x		322.4	-	-	-	11.9	-	-	-
	n	320×256	30.1	17.8	15.1	14.8	9.4	9.0	8.7	8.7
	s		33.2	21.4	17.2	14.9	10.5	9.0	9.0	8.9
	m		58.4	-	-	-	11.0	-	-	-
	l		91.5	-	-	-	11.2	-	-	-
	x		135.2	-	-	-	11.1	-	-	-
	n	160×128	29.0	13.5	12.1	8.8	8.9	8.7	8.6	8.6
	s		29.2	15.8	13.2	12.8	9.7	8.8	8.8	8.7
	m		41.8	-	-	-	10.4	-	-	-
	l		60.0	-	-	-	10.8	-	-	-
	x		81.2	-	-	-	11.0	-	-	-

**Table 4:** Accuracy metrics across precision levels and resolutions: box mAP50–95, pose mAP50–95 and mean OKS.

Model		Input	Box mAP50-95				Pose mAP50-95				mean OKS			
		Resolution	PT	FP32	FP16	INT8	PT	FP32	FP16	INT8	PT	FP32	FP16	INT8
YOLO11-pose	n	640×512	0.991	0.989	0.989	0.86	0.899	0.892	0.892	0.136	0.921	0.921	0.921	0.488
	s		0.991	0.99	0.99	0.904	0.903	0.893	0.894	0.165	0.923	0.923	0.923	0.491
	m		0.992	0.991	0.991	0.933	0.925	0.919	0.919	0.331	0.934	0.934	0.934	0.597
	l		**0.994**	0.993	0.993	0.935	0.93	0.928	0.927	0.193	0.935	0.935	0.935	0.499
	x		0.993	0.993	0.993	0.878	**0.937**	0.935	0.935	0.251	**0.94**	**0.94**	**0.94**	0.595
	n	320×256	0.989	0.989	0.989	0.954	0.866	0.867	0.867	0.407	0.908	0.908	0.907	0.672
	s		0.992	0.992	0.991	0.946	0.885	0.883	0.884	0.459	0.919	0.919	0.918	0.707
	m		**0.993**	**0.993**	**0.993**	0.926	0.92	0.919	0.919	0.469	0.932	0.932	0.932	0.724
	l		**0.993**	**0.993**	**0.993**	0.965	0.921	0.92	0.921	0.514	0.933	0.933	0.932	0.715
	x		**0.993**	0.992	0.992	0.957	**0.925**	0.922	0.921	0.326	**0.934**	**0.934**	**0.934**	0.636
	n	160×128	0.945	0.946	0.946	0.919	0.739	0.737	0.737	0.388	0.849	0.849	0.848	0.657
	s		0.982	0.979	0.979	0.94	0.842	0.84	0.84	0.53	0.898	0.898	0.898	0.744
	m		0.983	0.981	0.981	0.943	0.853	0.848	0.847	0.56	0.9	0.9	0.9	0.748
	l		**0.989**	0.988	0.988	0.971	0.871	0.87	0.87	0.554	0.912	0.912	0.912	0.756
	x		0.987	0.986	0.986	0.971	**0.888**	0.887	**0.888**	0.605	**0.918**	**0.918**	**0.918**	0.769
YOLOv8-pose	n	640×512	**0.993**	0.992	0.992	0.921	0.904	0.9	0.9	0.279	0.926	0.927	0.926	0.561
	s		0.992	0.991	0.991	0.917	0.935	0.928	0.928	0.401	0.942	0.942	0.942	0.688
	m		**0.993**	0.992	0.992	0.919	0.937	0.929	0.929	0.43	0.943	0.943	0.943	0.713
	l		0.992	0.991	0.991	0.898	0.942	0.939	0.938	0.301	0.944	0.944	**0.945**	0.616
	x		0.992	0.992	0.992	0.93	**0.943**	0.941	0.942	0.296	**0.945**	**0.945**	**0.945**	0.599
	n	320×256	0.988	0.988	0.988	0.939	0.871	0.866	0.867	0.395	0.911	0.911	0.911	0.646
	s		0.99	0.989	0.989	0.911	0.893	0.884	0.885	0.457	0.92	0.92	0.92	0.714
	m		**0.992**	0.991	0.991	0.952	0.904	0.899	0.899	0.461	0.927	0.927	0.927	0.695
	l		0.991	0.99	0.99	0.957	0.937	0.936	0.935	0.538	0.941	0.941	0.941	0.737
	x		0.991	0.99	0.99	0.971	**0.939**	0.937	0.937	0.401	**0.943**	**0.943**	**0.943**	0.667
	n	160×128	0.952	0.947	0.947	0.866	0.751	0.739	0.74	0.342	0.851	0.851	0.851	0.527
	s		0.973	0.967	0.967	0.853	0.809	0.793	0.792	0.41	0.882	0.882	0.882	0.668
	m		0.982	0.98	0.98	0.936	0.845	0.84	0.84	0.551	0.902	0.902	0.902	0.766
	l		**0.987**	**0.987**	**0.987**	0.941	0.874	0.874	0.873	0.598	0.912	0.912	0.912	0.737
	x		0.985	0.984	0.984	0.95	**0.886**	0.881	0.881	0.622	**0.919**	**0.919**	**0.919**	0.797

*Note:* Gray shading indicates the best precision mode for each model scale and resolution. Boldface indicates the best model scale within each resolution.

**Table 5:** Mean accuracy degradation relative to PyTorch. Results are averaged over five model scales (n–x) for each input resolution.

Pose	Input	ΔOKS			ΔPose mAP50–95		
Model	Resolution	FP32	FP16	INT8	FP32	FP16	INT8
v11	640×512	−0.000±0.001	−0.001±0.001	−0.327±0.081	−0.001±0.002	−0.002±0.002	−0.301±0.073
	320×256	−0.001±0.001	−0.002±0.001	−0.227±0.057	−0.002±0.002	−0.003±0.002	−0.206±0.051
	160×128	−0.001±0.001	−0.003±0.001	−0.165±0.042	−0.002±0.002	−0.004±0.002	−0.152±0.039
v8	640×512	−0.000±0.001	−0.001±0.001	−0.318±0.074	−0.001±0.002	−0.002±0.002	−0.287±0.066
	320×256	−0.001±0.001	−0.002±0.001	−0.213±0.051	−0.002±0.002	−0.003±0.002	−0.194±0.047
	160×128	−0.001±0.001	−0.003±0.001	−0.152±0.038	−0.002±0.002	−0.004±0.002	−0.141±0.035

*Note:* Background colors follow a heatmap-style gradient indicating the magnitude of mean accuracy degradation, ranging from smaller degradation (green) through moderate values (yellow) to larger degradation (red).

## Data Availability

The original data presented in this study are openly available in Mendeley at https://data.mendeley.com/datasets/64dhsznpfx/1 (accessed on 27 January 2026).

## References

[1] Latreche A., Kelaiaia R., Chemori A., Kerboua A. (2023). A new home-based upper-and lower-limb telerehabilitation platform with experimental validation. Arab. J. Sci. Eng..

[2] Clemente C., Chambel G., Silva D.C., Montes A.M., Pinto J.F., Silva H.P.D. (2023). Feasibility of 3D body tracking from monocular 2D video feeds in musculoskeletal telerehabilitation. Sensors.

[3] Aguilar-Ortega R., Berral-Soler R., Jiménez-Velasco I., Romero-Ramírez F.J., García-Marín M., Zafra-Palma J., Muñoz-Salinas R., Medina-Carnicer R., Marín-Jiménez M.J. (2023). Uco physical rehabilitation: New dataset and study of human pose estimation methods on physical rehabilitation exercises. Sensors.

[4] Gao Z., Chen J., Liu Y., Jin Y., Tian D. (2025). A systematic survey on human pose estimation: Upstream and downstream tasks, approaches, lightweight models, and prospects. Artif. Intell. Rev..

[5] Sykes E.R. (2025). Next-generation fall detection: Harnessing human pose estimation and transformer technology. Health Syst..

[6] Zhu Y., Xiao M., Xie Y., Xiao Z., Jin G., Shuai L. (2024). In-bed human pose estimation using multi-source information fusion for health monitoring in real-world scenarios. Inf. Fusion.

[7] Bustos N., Mashhadi M., Lai-Yuen S.K., Sarkar S., Das T.K. (2023). A systematic literature review on object detection using near infrared and thermal images. Neurocomputing.

[8] Zhang W., Li J., Tien P., Calautit J.K. (2025). Vision-based Occupancy Detection in Indoor Environments: A Comparison of Standard RGB and Thermal Cameras. J. Build. Eng..

[9] Guo Y., Chen Y., Deng J., Li S., Zhou H. (2022). Identity-preserved human posture detection in infrared thermal images: A benchmark. Sensors.

[10] Ficili I., Giacobbe M., Tricomi G., Puliafito A. (2025). From sensors to data intelligence: Leveraging IoT, cloud, and edge computing with AI. Sensors.

[11] Liu J., Du Y., Yang K., Wu J., Wang Y., Hu X., Wang Z., Liu Y., Sun P., Boukerche A. (2025). Edge-cloud collaborative computing on distributed intelligence and model optimization: A survey. arXiv.

[12] Cajas Ordóñez S.A., Samanta J., Suárez-Cetrulo A.L., Carbajo R.S. (2025). Intelligent edge computing and machine learning: A survey of optimization and applications. Future Internet.

[13] Zeng L., Huang C., Xie R., Huang Z., Guo Y., He L., Xie Z., Xing G. (2025). ThermiKit: Edge-Optimized LWIR Analytics with Agent-Driven Interactions. Proceedings of the 2025 ACM International Workshop on Thermal Sensing and Computing.

[14] Smith J., Loncomilla P., Ruiz-Del-Solar J. (2023). Human pose estimation using thermal images. IEEE Access.

[15] Chen I.C., Wang C.J., Wen C.K., Tzou S.J. (2020). Multi-person pose estimation using thermal images. IEEE Access.

[16] Zang Y., Fan C., Zheng Z., Yang D. (2021). Pose estimation at night in infrared images using a lightweight multi-stage attention network. Signal Image Video Process..

[17] Cormier M., Yi C.N.Z., Specker A., Blaß B., Heizmann M., Beyerer J. (2024). Leveraging Thermal Imaging for Robust Human Pose Estimation in Low-Light Vision. Proceedings of the Asian Conference on Computer Vision.

[18] Lupión M., Polo-Rodríguez A., Medina-Quero J., Sanjuan J.F., Ortigosa P.M. (2024). 3D Human Pose Estimation from multi-view thermal vision sensors. Inf. Fusion.

[19] Tambwekar A., Park B.K.D., Kusari A., Sun W. (2024). Three-Dimensional Posture Estimation of Vehicle Occupants Using Depth and Infrared Images. Sensors.

[20] Boldo M., De Marchi M., Martini E., Aldegheri S., Quaglia D., Fummi F., Bombieri N. (2024). Real-time multi-camera 3D human pose estimation at the edge for industrial applications. Expert Syst. Appl..

[21] Analia R., Forster A., Xie S.Q., Zhang Z. (2025). Privacy-Preserving Approach for Early Detection of Long-Lie Incidents: A Pilot Study with Healthy Subjects. Sensors.

[22] Wang Z., Sun H., Calautit J.K., Yang M., Chen J., Guan R., Darkwa J. (2025). A novel lightweight skeletal temporal model for real-time, computationally efficient recognition of occupant thermal adaptation behavior. Building Simulation.

[23] Kuzdeuov A., Zakaryanov M., Tleuliyev A., Varol H.A. (2025). OpenThermalPose2: Extending the Open-Source Annotated Thermal Human Pose Dataset With More Data, Subjects, and Poses. IEEE Trans. Biom. Behav. Identity Sci..

[24] Gad M.M., Gad W., Abdelkader T., Naik K. (2025). Personalized Smart Home Automation Using Machine Learning: Predicting User Activities. Sensors.

[25] Park J., Yang K.O., Park S., Choi J.W. (2025). Human Daily Indoor Action (HDIA) Dataset: Privacy-Preserving Human Action Recognition Using Infrared Camera and Wearable Armband Sensors. IEEE Access.

[26] Iadarola G., Mengarelli A., Iarlori S., Monteriù A., Spinsante S. (2025). RGB-D Cameras and Brain–Computer Interfaces for Human Activity Recognition: An Overview. Sensors.

[27] Cormier M., Specker A., Beyerer J. (2025). UPPET: Unified Pedestrian Pose Estimation in Thermal Imaging. Proceedings of the IEEE/CVF Conference on Computer Vision and Pattern Recognition Workshops (CVPRW).

[28] Chen Z., Yang J., Chen L., Li F., Feng Z., Jia L., Li P. (2025). RailVoxelDet: An Lightweight 3D Object Detection Method for Railway Transportation Driven by on-Board LiDAR Data. IEEE Internet Things J..

[29] You K., Gu Y., Shao H., Wang Y. (2026). A liquid-impulse neural network model based on heterogeneous fusion of multimodal information for interpretable rotating machinery fault diagnosis. Mech. Syst. Signal Process..

[30] Zhu Y., Lu W., Zhang R., Wang R., Robbins D. (2022). Dual-channel cascade pose estimation network trained on infrared thermal image and groundtruth annotation for real-time gait measurement. Med. Image Anal..

[31] Chen L., Sun Q., Xu Z., Liao Y., Chen Z.D. (2025). A low-resolution infrared gesture recognition method combining weak information reconstruction and joint training strategy. Digit. Signal Process..

[32] Wang Y., Wu Y., Ogai H., Dong Y., Tateno S. Edge-Oriented Person Activity Recognition and Alert System for Ensuring Safety of Independent Elderly at Home. Proceedings of the 2025 8th International Conference on Information Communication and Signal Processing (ICICSP).

